# Putative Multifunctional Signature of Lung Metastases in Dedifferentiated Chondrosarcoma

**DOI:** 10.1155/2012/820254

**Published:** 2012-02-16

**Authors:** Sergey Malchenko, Elisabeth A. Seftor, Yuri Nikolsky, Susan L. Hasegawa, Sean Kuo, Jeff W. Stevens, Stas Poyarkov, Tatiana Nikolskaya, Tamara Kucaba, Min Wang, Hakim Abdulkawy, Thomas Casavant, Jose Morcuende, Joseph Buckwalter, Raymond Hohl, Barry DeYoung, Kemp Kernstine, Maria de Fatima Bonaldo, Mary J. C. Hendrix, Marcelo B. Soares, Vera Maria F. C. Soares

**Affiliations:** ^1^Cancer Biology and Epigenomics Program, Children's Memorial Research Center, and Department of Pediatrics, Northwestern University Feinberg School of Medicine, 2430 N. Halstead Street, Chicago, IL 60614, USA; ^2^GeneGo, Inc., 500 Renaissance Drive, No. 106, St. Joseph, MI 49085, USA; ^3^Department of Pathology and Laboratory Medicine, Children's Memorial Hospital 2300 Children's Plaza, Chicago, IL 60614, USA; ^4^Center for Bioinformatics and Computation Biology, University of Iowa, 5316 Seamans Center, Iowa City, IA 52242, USA; ^5^Department of Orthopaedics and Rehabilitation, University of Iowa, 200 Hawkins Drive, Iowa City, IA 52242, USA; ^6^Vavilov Institute for General Genetics, Russian Academy of Sciences, 3 Gubkina Street, Moscow 117093, Russia; ^7^Department of Urology, University of Iowa, 200 Hawkins Drive, Iowa City, IA 52242, USA; ^8^Department of Hematology/Oncology, University of Iowa, 200 Hawkins Drive, Iowa City, IA 52242, USA; ^9^Department of Surgical Pathology, University of Iowa, 200 Hawkins Drive, Iowa City, IA 52242, USA; ^10^Lung Cancer and Thoracic Oncology Program, Beckman Research Institute, City of Hope, 1500 East Duarte Road, Duarte, CA 91010, USA; ^11^Department of Pediatrics, University of Iowa, 200 Hawkins Drive, Iowa City, IA 52242, USA

## Abstract

Chondrosarcomas are among the most malignant skeletal tumors. Dedifferentiated chondrosarcoma is a highly aggressive subtype of chondrosarcoma, with lung metastases developing within a few months of diagnosis in 90% of patients. In this paper we performed comparative analyses of the transcriptomes of five individual metastatic lung lesions that were surgically resected from a patient with dedifferentiated chondrosarcoma. We document for the first time a high heterogeneity of gene expression profiles among the individual lung metastases. Moreover, we reveal a signature of “multifunctional” genes that are expressed in all metastatic lung lesions. Also, for the first time, we document the occurrence of massive macrophage infiltration in dedifferentiated chondrosarcoma lung metastases.

## 1. Introduction

Chondrosarcoma is the second most common malignant skeletal tumor. One of the subtypes of chondrosarcoma—dedifferentiated chondrosarcoma—is a high-grade pleomorphic noncartilaginous sarcoma, arising within a low-grade chondrosarcoma [1]. The median survival time for the dedifferentiated chondrosarcoma patients is 7.5 months [2]. Despite the fact that the process of metastatic dissemination depends upon a number of highly coordinated rate limiting steps [3, 4], it is remarkable that lung metastases develop within a few months of diagnosis in 90% of dedifferentiated chondrosarcoma patients. 

There is an increasing body of evidence pointing to the involvement of stem-like cells in the process of sarcoma's metastatic dissemination [5–7]. In an attempt to elucidate the observed high frequency of dedifferentiated chondrosarcoma metastases, we developed a hypothesis that dedifferentiated chondrosarcoma-initiating cells might have some characteristics of stem-like cells. We further hypothesized that such dedifferentiated chondrosarcoma-initiating cells might exhibit at least two predominant features: multipotentiality, as one of the stem cell characteristics, which would explain pleomorphic histology of dedifferentiated chondrosarcoma [1, 8], and “multifunctionality”-expression of a set of “multifunctional” genes, which would explain fast progression of dedifferentiated chondrosarcoma through all the rate-limiting steps required for metastatic dissemination.

Based on the hypothetical multipotentiality of dedifferentiated chondrosarcoma-initiating cells, we suspected that there would be a high degree of heterogeneity among metastases of a single patient. This would explain at least in part the notorious untreatability of dedifferentiated chondrosarcoma lung metastases. Indeed, by using Serial Analysis of Gene Expression (SAGE) we found a high degree of heterogeneity at the gene expression level among the different lung metastases from a single dedifferentiated chondrosarcoma patient. Also, we found a signature of “multifunctional” genes in all the metastatic lesions. Remarkably, most of these genes are not only known to be involved in metastatic dissemination in other tumor types but also to be expressed in mesenchymal stem cells. Analysis of this gene signature predicted the occurrence of a leukocyte infiltration in these metastases. Indeed, for the first time, we have documented the occurrence of a massive macrophage infiltration in the dedifferentiated chondrosarcoma lung metastases.

## 2. Materials and Methods

### 2.1. Patients Involved in This Study (IRB Number 199703227)

Patient A was a female diagnosed at 45 years of age with a pelvic dedifferentiated chondrosarcoma. Following surgical resection, the patient received pelvic radiation therapy at the site of the primary tumor (66 Gg—35 sessions). Two months following resection, the patient was diagnosed with lung metastases. The patient received chemotherapy (Adriamycin (30 mg/m^2^—day 1, 30 mg/m^2^—day 2) and Cis-platinum (120 mg/m^2^—day 1, 200 mg/m^2^—day 2)), which was administered once every three weeks for a period of nine weeks. Following chemotherapy the patient underwent excision of the metastatic lesions.

Patient B was a male diagnosed at twenty eight years of age with a recurrent intrapelvic chondrosarcoma. He was initially admitted to the hospital and diagnosed with intrapelvic chondrosarcoma Grade I, which was surgically resected. Over the next 10 years, the patient experienced local recurrences nearly every year. During this period the recurrent tumor progressed from histologic grade I to grade II. Remarkably, no evidence of distant metastatic disease has been reported to date.

### 2.2. Histology and Immunohistochemistry

Four micron-thick sections were prepared from formalin-fixed, paraffin-embedded tissue obtained from the pelvic resection and initial lung metastasis of patient A and two separate recurrences from patient B. Sections were either stained with hematoxylin and eosin using standard procedures or were subject to immunohistochemical staining. Immunohistochemical stains were performed using the Envision+ Dual Link System Peroxidase Kit (Dako) using primary antibodies directed against CD68 (monoclonal KP1, Dako, dilution 1 : 80) or CD15 (monoclonal MMA, Cell Marque, dilution 1 : 50). Antigen retrieval techniques involved digestion in proteinase K for five minutes for the KP1 antibody or pressure cooking for 30 minutes for the MMA antibody. The sections were then counterstained with hematoxylin. Appropriate positive control tissues were used, and antibody was omitted in negative control slides.

### 2.3. Establishment and General Maintenance of Metastatic and Nonmetastatic Cell Lines

Fresh tissue samples from patient A's metastatic lesions were divided into 1 mm^3^ portions and incubated at 37°C for 30 minutes with testicular hyaluronidase type IA (Sigma-Aldrich) (0.1%, w/v) in growth medium that contained heat inactivated fetal bovine serum (Life Technologies Gibco) (10%), Dulbecco's modified Eagle medium, glucose (4.5 gm/L), and HEPES buffer (25 mM) solution. The tissue samples were then further treated with collagenase type 1A (Sigma-Aldrich) (0.05%, w/v) and dispase (Life Technologies Gibco) (0.05%, w/v) in growth medium containing gentamycin (50 ug/mL), incubated at 37°C in a CO^2^ (5%) incubator for 21 hours. Monodispersed cells were obtained following filtration of the enzyme-treated tissue through 70 mkm and 40 mkm screens and frozen in diemethylsulfoxide/heat-inactivated fetal bovine serum (10%/90%, v/v). Metastatic cell lines (Met.-cell lines) were established from these cells after culturing in RPMI-1640 supplemented with 10% FBS and 0.1% gentamycin sulfate (Gemini Bioproducts). Cells were cultured through four doublings to yield 2 × 10^7^ cells.

Fresh tissue samples from patient B's recurrent tumors were divided by gross analysis into the cartilaginous and fibro-cartilaginous samples for tissue-cell isolation. The protocol described previously was used for establishment and maintenance of the two resulting cell lines (NM-cell lines).

All the cell lines were routinely screened for mycoplasma contamination using a PCR-based ELISA detection assay (Roche). Cell manipulations were always performed on 80–90% confluent cultures for consistency.

### 2.4. Invasion Assay

The Membrane Invasion Culture System (MICS) chamber was used to evaluate the degree of tumor cell invasion through ECMs *in vitro* as described previously [9]. Percent invasion was corrected for proliferation and calculated as total number of invading cells from lower chamber divided by the total number of cells seeded in the upper chamber ×100. Six wells were dedicated to test each cell line (Supplemental Table 5c) per experiment, and each experiment was repeated at least 3 times. The data generated from these studies were statistically analyzed for “one-way analysis of variance” using the statistical package (ANOVA analysis) of the Microsoft Excel spreadsheet program.

### 2.5. RNA Extraction

Total RNA for RT-PCR was isolated from the cultured cells using RNazol B (Biotecx Laboratories, Inc., Houston, TX, USA) according to the manufacturer's instructions. PolyA^+^ RNA for the SAGE library constructions was isolated from the cultured cells using Dynabeads mRNA DIRECT kit (Dynal A.S, Oslo, Norway) according to the manufacturer's instructions.

### 2.6. SAGE Library Construction

Double-strand cDNA was synthesized from RNA isolated from Met. 1–5 and NM. 1 and 2 (Table 1) as it has been previously described [10]. The SAGE protocol utilized for library construction in this study was a modification of a previously described procedure [11]. Specifically, we modified the SAGE procedure by using T4 DNA Polymerase to make blunt end concatameres, and subsequently cloned them into a blunt ended pUC18 plasmid. After electroporation and overnight growth on plates, we utilized a sequencing protocol without the PCR size selection step, required in the original protocol. We used SAGE 2000 software (http://www.sagenet.org/) to extract the SAGE tags and to calculate the frequency at which each tag is seen within a SAGE library. More than 77,000 tags were sequenced from each library and the size of each library was normalized to 100,000 tags. After the normalization, all the tags were mapped to NCBI's UniGene clusters, using the SAGE GENIE database [12].

We have combined and averaged cluster sizes of identical tags from the normalized NM. 1 and NM. 2 libraries in order to create a virtual NM-library of the nonmetastatic tumor.

All SAGE libraries were submitted to the NCI Cancer Genome Anatomy Project SAGE library collection. Pairwise comparisons of the normalized SAGE libraries were done by applying the *z*-test [13]. The normalized SAGE data were used as the input for the Gene Spring 7.3.1 program (Silicon Genetics, Redwood City, CA, USA), which allows multifilter comparisons and generation of conditional (hierarchical) trees.

### 2.7. DNA Sequencing

Sequencing reactions were performed with the ABI PRISM dRhodamine terminator cycle sequencing kit and −40 M13 Forward primers. Reaction products were electrophoresed on an ABI PRISM 3700 DNA Analyzer.

### 2.8. Datasets for the Functional Analysis

#### 2.8.1. Genes Commonly Expressed in Metastatic Lung Lesions of Patient A

We selected the genes (defined as unique EntrezGene identifiers) that were expressed in all five libraries, derived from the Met.-cell lines (see Section 2.6). This dataset contained 1,488 “common” genes, including 59 genes of ribosomal proteins (see below).

#### 2.8.2. Genes Expressed in the Nonmetastatic Tumor of Patient B

This dataset contained 3,249 genes that were commonly expressed in the two libraries composing the virtual NM.-library (see Section 2.6).

#### 2.8.3. Literature-Based List of Genes Previously Associated with Metastasis

Genes in this list were annotated by GeneGo based on PubMed searches. The list contained 339 genes associated with the process of metastatic dissemination (Table 2 see in Supplementary Material available online at doi: 10.1155/2012/820254).

#### 2.8.4. Genes with Differential Expression in the Metastases

Genes in this list were expressed in all five libraries derived from Met.-cell lines and differentially expressed in the virtual NM.-library. This dataset contained 158 differentially expressed genes: 88 upregulated and 70 downregulated in all the Met.-cell lines. Interestingly, 26 of the 70 downregulated genes encode ribosomal proteins (Supplementary Table 2).

#### 2.8.5. Genes from “Metastasis-Related” Processes

The list contained genes annotated by GeneGo from eight functional categories of relevance to metastasis, namely, cell motility, (126 genes), cell adhesion (483 genes), chemotaxis (125 genes), blood coagulation (81 genes), cell proliferation (302 genes), ECM remodeling (99 genes), angiogenesis (66 genes), and antiapoptosis (118 genes).

#### 2.8.6. “Multifunctional” Genes

The criterion utilized for selection was “multifunctionality”; that is, the selected proteins should belong to at least two of the 8 functional categories compiled by GeneGo (see Section 2.8.5). A total of 161 proteins were selected and herein coined, “multifunctional” grid. Interestingly, 36 of such “multifunctional” genes are also in the list of genes from the metastasis associated literature (see Section 2.8.3).

### 2.9. Gene List Enrichment Analysis

First, the lists of significantly differentially expressed genes among different metastases were generated by conducting pairwise comparisons between the genes expressed in UIFK0-Met. 1 (4,205 clusters) and those expressed in each of the other metastases, that is, Met. 2–5. Next, to evaluate the enrichment of the “metastasis associated” and the “multifunctional” genes in such lists of significantly differentially expressed genes, a simulation of randomly chosen clusters was carried out. For each iteration of the simulation, 105 or 52 clusters were randomly chosen from the total 4,205 clusters from UIFK0-Met. 1 (see Section 3). Out of these 105 or 52 randomly chosen clusters, the numbers of clusters that appeared in the lists of significantly differentially expressed genes were determined and the corresponding ratios for these clusters were calculated. A total of 100,000 iterations were performed and the average ratios were calculated. These average ratios were used as baseline levels in order to compare with the representations of the specific 105 “metastasis-associated” and the 52 “multifunctional” genes. In order to determine the significance of the enrichment, one sample binomial test was carried out for each of the corresponding comparisons. The R statistical package and Microsoft Excel software were used to perform these statistical calculations.

### 2.10. Functional Analysis of SAGE Data

 For functional analysis, we applied an integrated human data mining suit—MetaCore (GeneGo, Inc.)—which has been described elsewhere [14].

### 2.11. Real-Time PCR

Real-time quantitative reverse transcription-polymerase chain reactions (QRT-PCRs) were performed using an IQ5 Cycler (Bio-Rad Laboratories, USA) according to the manufacturer's instructions. Primers were designed using the Primer Express program version 1.5 (Applied Biosystems, CA, USA) and obtained from Integrated DNA Technologies (Coralville, IA, USA; Supplementary Table 1). The specificity of the primers was documented by RT-PCR and resulted in a single product with the desired length. cDNAs were constructed using IScript cDNA synthesis kit (Bio-Rad Laboratories, USA) according to the manufacturer's instruction. Reactions were performed using IQ SYBR Green Supermix kit (Bio-Rad Laboratories, USA) according to the manufacturer's instruction. Each reaction was performed in triplicate, using 250 nM primers, cDNA sample corresponding to 0.25 ng of total RNA, in a total volume of 25 *μ*L. 100 nM primers for 18S rRNA from the TaqMan Ribosomal RNA Control Reagents kit (Applied Biosystems, CA, USA) were used as a reference for each of the cDNA samples. The PCR conditions were as follows: one cycle at 95°C for 3 min, 34 cycles at 95°C for 30 sec, 55°C for 30 sec, 72°C for 30 sec, followed by a melting curve from 55°C to 95°C. A standard curve was generated using serial dilutions of the template cDNA for the reference gene and for each gene of interest. The efficiencies of amplification for each gene were calculated using software provided by the IQ5 Cycler. An equimolar pool of cDNA samples from two NM.-cell lines was used as a calibrator. We used the Pfaffl method to calculate the relative gene expression levels (Bio-Rad Laboratories, Inc. Real-Time PCR Applications Guide p-43).

## 3. Results

### 3.1. Initial Histological Analysis of the Primary Tumor and the Metastases

**Figure 1:**
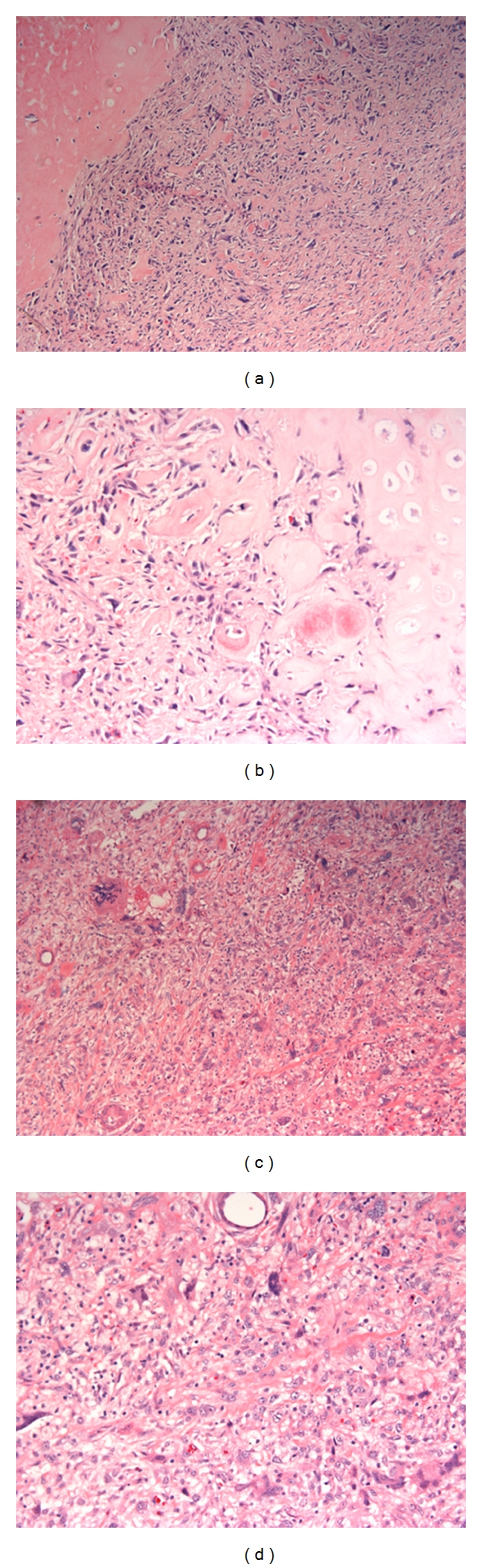
(a) The pelvic resection specimen of dedifferentiated chondrosarcoma demonstrated low-grade component (left) abruptly juxtaposed to high-grade dedifferentiated component (right) (10x, H&E). (b) Focal osteoid formation was present within the dedifferentiated component (20x, H&E). (c) The lung metastases consisted entirely of the dedifferentiated component of the primary tumor (10x, H&E). (d) Focal osteoid formation was noted within the metastatic tumor (20x, H&E).

A pelvic resection specimen from the patient A (Section 2) demonstrated the typical histological features of a dedifferentiated chondrosarcoma (Figure 1(a)). Areas of low-grade chondrosarcoma were abruptly juxtaposed to distinct areas of high-grade spindle cell sarcoma, characterized by a cellular proliferation of highly pleomorphic spindle to stellate cells with anaplastic and hyperchromatic nuclei. Focal osteoid matrix formation was noted within the dedifferentiated foci (Figure 1(b)). In contrast to the primary tumor specimen, the metastatic foci consisted entirely of the high-grade dedifferentiated portion of the tumor, and no evidence of the low-grade chondrosarcoma component was identified (Figure 1(c)). Similarly to the primary dedifferentiated component, focal osteoid formation was present within the metastases (Figure 1(d)).

### 3.2. Invasion Assay

 To evaluate the degree of tumor cell invasion of the metastatic and nonmetastatic cell lines we used the MICS chamber as described previously [9]. Met. 2- and Met. 3-cell lines were excluded from the assay due to limited amount of cells. All the remaining metastatic cell lines showed significantly higher percentage of invading cells, after 24 hours in the invasion assays, in comparison with both NM cell lines (Supplementary Table 5c).

### 3.3. Molecular Analyses of the Lung Metastases

**Figure 2:**
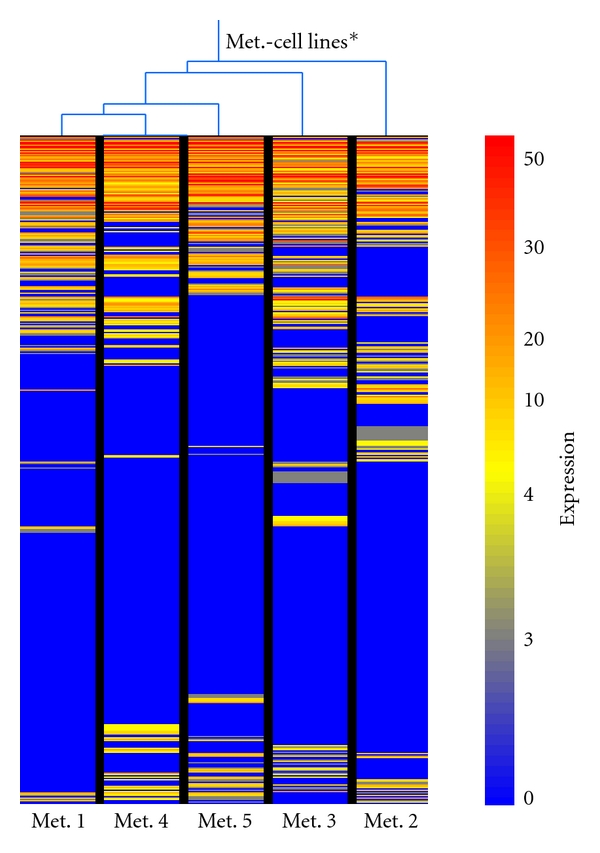
Hierarchical clustering analysis of the SAGE libraries, generated from the Met.-cell lines, using the standard correlation algorithms (GeneSpring). *Cluster size ≥ 2.

Aiming to identify potential differences in expression among the five metastases, we isolated RNA from the five cell lines that were established from each of the metastases and SAGE libraries were constructed. In order to identify relationships among the Met. cell lines, we conducted a hierarchical clustering analysis [15] using GeneSpring with the standard correlation algorithm. As it can be seen in Figure 2, Met. 1 and Met. 2 are the farthest apart; hence they exhibit the most difference in expression pattern.

Next we conducted pairwise comparisons between the genes expressed in Met. 1 (4,205 genes) and those expressed in each of the other metastases, that is, Met. 2–5 (Supplemental Table 4A). This was done with the goal of identifying statistically significant differences and similarities in gene expression among the five lesions. We then derived a set of “metastases-associated” genes that were expressed in Met. 1 (105 genes) by intersecting the genes in Section 2.8.3 (“metastasis-associated” genes) with the genes expressed in Met. 1. Similar pairwise comparisons were then performed between the subset of 105 “metastasis-associated” genes identified in Met. 1 and those expressed in each of the other metastases (Supplementary Table 4B). Last, we derived a list of “multifunctional” genes expressed in Met. 1 (52 genes)—by intersecting the genes in Section 2.8.6 (“Multifunctional” genes) with those expressed in Met. 1—and performed pairwise comparisons between them and the genes expressed in each of the other metastases (Supplementary Table 4C).

The results from these sets of pairwise comparisons show that the percentage of differentially expressed genes is greater in the subset of 105 “metastasis associated” genes and in the subset of 52 “multifunctional” genes (*P* < 0.001 for all four comparisons) (Supplementary Tables 4B and C) than the respective percentages of differentially expressed genes when all the genes that are expressed in the metastases are compared. The aforementioned *P* values were calculated based upon computational simulation analyses of 105 and 52 randomly chosen clusters (data not shown).

To clarify that the differentially expressed genes are not derived from the low copy number gene population [10], we further assessed whether there was a bias in the cluster sizes of SAGE tags corresponding to transcripts that were significantly differentially expressed relative to those of the transcripts that did not exhibit differential expression in the metastases. Overall, significantly differentially expressed genes tended to have larger cluster sizes (SAGE tag average mean value 27.5) than those with no significant difference in expression (SAGE tag average mean value 6.77). Similar trends were also observed for the “metastasis-associated” and for the “multifunctional” genes; that is, SAGE tags corresponding to significantly differentially expressed genes tended to have larger cluster sizes (SAGE tag average mean value 43.27 and 59.1, resp.), in comparison to those with no significant difference in expression (SAGE tag average mean value 17.74 and 13.04, resp. (Supplementary Table 3)).

### 3.4. Analysis of “Multifunctional” Genes Expressed in the Lung Metastases

**Figure 3:**
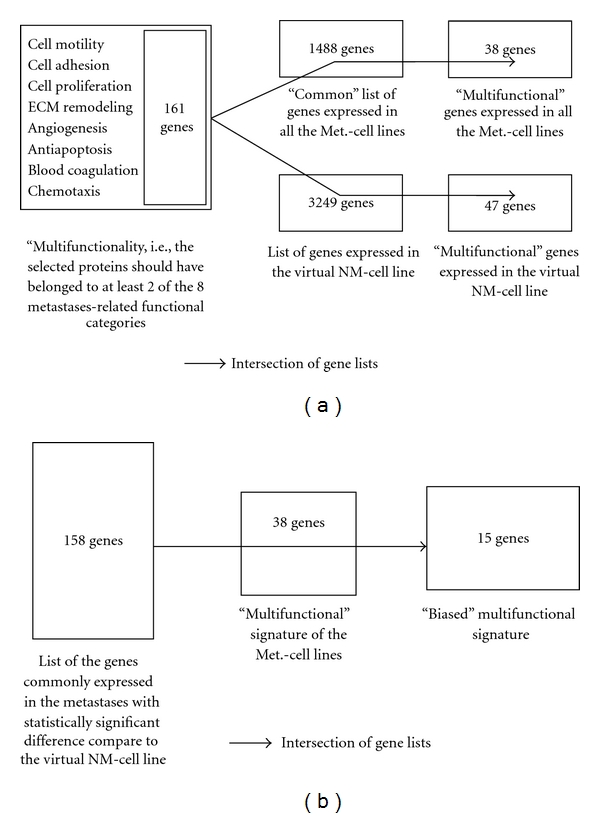
(a) Identification of “multifunctional” signatures. (b) Identification of “biased multifunctional” signature.

In order to identify a “multifunctional” gene signature in the Met. cell lines, we intersected “Section 2.8.6” (“multifunctional” grid—161 genes) with “Section 2.8.1 (commonly expressed genes in the Met.-cell lines-1488 genes). This analysis revealed 38 “multifunctional” genes, herein referred to as the “multifunctional signature” of the Met.-cell lines (Figure 3(a), Supplementary Table 2).

Interestingly, we found a number of genes in this multifunctional signature (PLAU, CCL2, IL8, CXCL1, CD44), which had been previously shown to be involved in the recruitment of leukocytes [16–19]. Based on this observation, we suspected that recruitment of leukocytes might be involved in the process of metastatic dissemination in dedifferentiated chondrosarcoma (see the following). In parallel, we intersected “Section 2.8.6” with “Section 2.8.2” (genes expressed in the virtual NM-cell line—3249 genes) and found 46 “multifunctional” genes (Figure 3(a), Supplementary Table 2). In total, there were fifty five genes in the two “multifunctional” signatures combined: 8 genes were uniquely expressed in the Met.-cell lines (average mean value 32.7), and 17 genes were uniquely expressed in the virtual NM-cell line (average mean value 23.9). It is conceivable that an “effective” combination of “multifunctional” genes might exist, in that they may act synergistically and provide functional redundancy to promote or to facilitate the process of metastatic dissemination (see the following).

Next, we determined which of the 38 genes in the “multifunctional signature” of metastasis were differentially expressed in the nonmetastatic tumor, by intersecting it with the genes in “Section 2.8.4” (158 genes with significantly altered expression in the metastases compared to the nonmetastatic tumor). This analysis revealed 15 differentially expressed “multifunctional” genes—herein referred to as the “biased multifunctional signature”—seven of which were differentially expressed while the remainder eight were uniquely expressed in the metastases (Figure 3(b), Table 2). Interestingly, only 10.6% of the genes commonly expressed in the Met.-cell lines were differentially expressed in the virtual NM-cell line (158/1488 commonly expressed in the metastases). In contrast, 39.5% of “multifunctional” genes commonly expressed in the Met.-cell lines were found to be differentially expressed in the virtual NM-cell line (15/38 multifunctional genes) (Figure 3(b)).

The differential expression of a subset of genes in the “biased multifunctional signature” was validated by quantitative real-time PCR (Supplementary Table 1). We used the 15 genes of the “biased multifunctional signature” as an input list for generation of biological networks [14] (Supplementary Figure 1).

### 3.5. Immunohistochemistry of the Lung Metastasis

**Figure 4:**
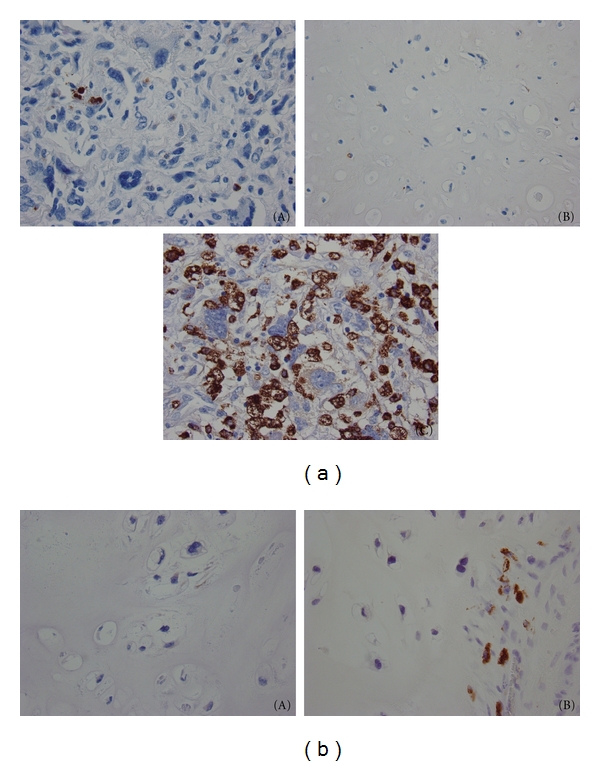
(a) CD68 staining demonstrates rare intratumoral macrophages in both the high-grade (A) and low-grade (B) components of the primary tumor from the patient A, whereas numerous intratumoral macrophages were present throughout the lung metastases (C) (40x, CD68). (b) (A) Stains for CD68 reveal very few intratumoral macrophages within a primary low-grade chondrosarcoma from the patient B (40x, CD68). (B) CD68 stains of the local recurrence highlight a few macrophages in the adjacent fibroconnective tissue but no intratumoral macrophages (40x, CD68).

Based upon our analysis of the “multifunctional signature” of metastasis, as aforementioned, we suspected that leukocyte infiltration might have been involved in the metastatic dissemination of patient's A dedifferentiated chondrosarcoma, a phenomenon that has been documented to occur in different types of tumors, including sarcomas [20–23]. Hence, we decided to stain sections of the primary tumor and of all five metastases of patient A as well as sections of the primary tumor obtained from patient B, with antibodies against CD68, a macrophage-specific antigen, and CD15, to detect neutrophils. Both low- and high-grade sections of patient A's primary tumor contained only rare intra-tumoral macrophages (Figure 4(a), panels A and B). In contrast, immunohistochemical stains of all metastatic lung lesions analyzed in this study showed a massive macrophage infiltrate (Figure 4(a)(C)). CD68-positive cells were fairly evenly distributed throughout the metastatic nodules and the cell density averaged approximately 180 per 40x high-power field. In contrast to the metastatic tumors, the locally recurrent, nonmetastatic tumor from patient B contained only few macrophages in the adjacent interstitial tissue and no significant intratumoral macrophages in either the original or recurrent tumor (Figure 4(b), panels A and B). Immunohistochemical stains for CD15 showed no significant intratumoral neutrophilic infiltrate within either the primary tumor or in the metastatic nodules of patient A, as well as in the nonmetastatic tumor of patient B (data not shown).

## 4. Discussion

As expected, the metastatic foci consisted entirely of the high-grade dedifferentiated portion of the tumor, with no evidence of the low-grade chondrosarcoma component. The cellular compositon of the metastases correlates with the results of the invasion assay, where metastatic cell lines showed significantly higher percentage of invading cells in comparison with both NM cell lines.

Despite the fact that the metastatic cell lines were generated from the dedifferentiated cells, they exhibited expression of a number of genes that are considered to be chondrocyte markers [24–33] (Supplementary Table 5a). As expected, however, the majority of the chondrocyte markers were downregulated in the metastatic cell lines in comparison with the two NM cell lines, consistent with the status of dedifferentiation. Additionally, we found expression of genes such as Collagen, type I, alpha 1 (COL1A1), Exostoses (multiple) 1 (EST1), Exostoses (multiple) 2 (EXT2), Vimentin (VIM), and Osteoprotegerin (TNFRSF11B), which are known to be involved in development of conventional chondrosarcomas [34–38] (Supplementary Table 5a).

Despite the limitations arising from the fact that no samples were available for analysis of the primary tumor, and that only restricted amounts of bulk tissue could be obtained from the metastatic lesions, our findings have shed some light to the molecular mechanisms underlying metastasis in dedifferentiated chondrosarcoma.

For the first time we documented a high heterogeneity at the gene expression level among individual lung metastases of a dedifferentiated chondrosarcoma patient. The heterogeneity is even greater for the “metastasis associated” and the “multifunctional” genes (Supplementary Table 4A–C), thus suggesting that it is indeed of “functional nature”.

There is a possibility that the high degree of heterogeneity observed among the metastases might be due to the manipulations that they were subjected to after resection from the patient. We believe this not to be the case because the five metastases were treated identically and, importantly, their cells were cultured under the same conditions and were only allowed to go through four doublings. Moreover, all five cultures were initiated at the same time, following the same procedures and utilizing the same reagents. Accordingly, it is noteworthy that of the 59 genes encoding ribosomal proteins found to be expressed in each of the five metastases, there is a group of 26 genes that were consistently downregulated relative to the virtual NM-cell line (Supplemental Table 2). Such consistent downregulation of the ribosomal proteins in the metastases serves to validate both the experimental and the statistical methods utilized in this study.

There is also the possibility that the heterogeneity in gene expression observed among the metastases might be due to occurrence of different aberrations in the karyotypes of their cells. However, we did not find any significant karyotype aberrations in the Met.-cell lines, except for Met. 5, in which two translocations t (5; 13) (q11.2; q32) and t (17; 18) (q23; p11.31) were identified in about 25% of the cells (data not shown; Dr. Shivanand R Patil, personal communication).

Lastly, the heterogeneity of expression might have resulted from the fact that each of the five lung lesions had a different “traveling history”. They might have been derived from different parts of the primary tumor and hence exposed to different microenvironments, which may also have occurred during lung colonization. It is conceivable that such a high level of heterogeneity observed among the metastases might contribute at least in part to their notorious untreatability.

Remarkably, most of the genes in the “biased multifunctional signature” are known to be involved in metastatic dissemination in different types of cancer. In particular, upregulation of urokinase (PLAU) was associated with an increased rate of metastasis and a decreased metastasis-free survival in 114 cases of chondrosarcoma of bone [39]. Our results, which revealed upregulation of PLAU and of tissue plasminogen activator (PLAT) in all Met.-cell lines, are in accordance with those reported by Häckel and colleagues [40]. They found that the high-grade dedifferentiated components of the tumors—but not the low-grade components of the same tumors, nor conventional chondrosarcomas-exhibited robust, diffuse coexpression of PLAU and PLAT. Other genes such as IL8, ITGB1, MSN, TFPI2, CAV1, and TGFB1 are also in Section 2.8.3 “metastasis associated genes” (Table 2, Supplementary Table 2) [41–47]. Furthermore, all the genes in the network shown in (Supplementary Materials Figure 1), with the exception of AP-1, are “multifunctional” and expressed in the Met.-cell lines. It is noteworthy that three of these genes—FN1, ILK, and CD44—are differentially expressed in four of the five Met.-cell lines, relative to the virtual NM-cell line. FN1, CD44, and CTNNB1 (beta-catenin) are also in the list of “metastasis-associated genes” (Supplementary Table 2) [19, 48–52]. Minn and colleagues [53] found MMP1, CXCL1, and TNC among 18 of the most significant (*P* < 0.05) genes in a lung metastasis signature. All three are in the “multifunctional” signature of dedifferentiated chondrosarcoma lung metastases—MMP1 and CXCL1 were significantly differentially expressed in 4 out of 5 metastases; TNC was differentially expressed in all 5 metastases and it is also a component of the “biased” signature). The authors also showed that combinations of MMP1 and CXCL1 could synergistically enhance lung colonization. Also, certain genes in the “biased” signature of dedifferentiated chondrosarcoma lung metastases, such as CCL2 and IL-8, were found to be significantly upregulated in primary tumors of nonsmall cell lung carcinoma with known history of lung metastases [54]. PLAU, another gene in the dedifferentiated chondrosarcoma- “biased” signature, was found to be involved in dissemination of bladder cancer lung metastases [55].

Noteworthy, majority of the genes from the network (Supplementary Figure 1)—PLAU, PLAT, TFPI2, ITGB1, CCL2, IL8, Cav1, FN1, ILK, and CD44—are known to be expressed in mesenchymal stem cells (MSCs) [56–61], as well as in other adult stem cells. Moreover, around 36% of the genes differentially expressed during chondrogenic differentiation of MSC [62] also had altered expression in the dedifferentiated chondrosarcoma metastases (27/74 genes differentially expressed in MSC) (Supplementary Table 5b). Some of these genes were found to be expressed at high level in both studies. Interestingly, the majority of the downregulated genes listed in this table are considered to be chondrocyte markers (AGC1, COL1a1, COMP, SPARC, Col3a1, BGN, FMOD) [24–27]. Hence, as expected, they did become downregulated during the dedifferentiation of chondrosarcoma and/or upregulated during the chondrocytic differentiation of MSC. FN1 was upregulated during the chondrogenic differentiation of MSC but in our study it was upregulated in the metastases (Supplementary Table 5b). In concordance with our data, FN1 was also upregulated in different metastatic chondrosarcomas [28].

Nonetheless, it remains to be determined if stem-like cells are indeed involved in the process of metastatic dissemination in dedifferentiated chondrosarcomas, and if “multifunctional” genes enable the rate-limiting steps of metastatic dissemination.

Although unlikely, it is conceivable that the fact that patient A had already been exposed to cytotoxic chemotherapy when the lung lesions were resected might have influenced our results. We find it to be unlikely because—with the exception of vinculin—none of the genes in the “biased signature” was upregulated in a SAGE library derived from the metastasis-free lung tissue sample obtained from the same dedifferentiated chondrosarcoma patient (Malchenko, S. and Soares, MB, personal communication), relative to the nonmetastatic tumor. Moreover, in other large-scale gene expression studies, none of the genes in the “biased multifunctional signature” exhibited altered expression upon exposure to a similar treatment regimen [63, 64].

This is the first report of a macrophage infiltration in lung metastases of dedifferentiated chondrosarcoma. It has been shown that macrophages, derived from circulating monocytes, represent a major component of the leukocyte infiltration in a tumor microenvironment [20]. An increase in the density of tumor-associated macrophages (TAMs) was correlated with poor prognosis in the majority of clinical studies in different types of cancer, including sarcomas [20–22]. Also, tumor overexpression of macrophage chemoattractants has been shown to correlate with poor prognosis [23]. Notwithstanding these findings, we cannot exclude that the observed macrophage infiltration represents either a specific antitumor defense mechanism or a general “physiological” reaction on local microenvironmental changes caused by the metastases [65]. However, the evenly high macrophage density that was observed all throughout the metastatic nodules is arguably not consistent with such interpretation.

Functional redundancy plays an important role in cancer development [66]. It is conceivable that coexpression of certain genes in the “multifunctional” signature of dedifferentiated chondrosarcoma metastases—such as CD44, PLAU, CXCL1, CCL2, and IL8—might increase the level of functional redundancy in the process of macrophage recruitment to the dedifferentiated chondrosarcoma metastases [16–19, 67]. Functional synergy might also play an important role in cancer development. Quite intriguing are the examples of synergy between some of these “multifunctional” genes—CCL2 and IL8, MMP1 and CXCL1 [16, 53, 68], and PLAT and PLAU [69]—suggesting that the synergizing capacity of “multifunctional” genes might bear significance to the process of metastatic dissemination. Unfortunately, due to the restricted amount of tissues, as it was mentioned previously, we did not have an opportunity to directly correlate protein expression of the “multifunctional” genes with macrophage recruitment in the metastatic lesions. Also, since there is no *in vivo* model of dedifferentiated chondrosarcoma lung metastasis, we did not have an option to analyse the involvement of the “multifunctional” genes in the process of metastatic dissemination experimentally.

In summary, we provide evidence for the first time of high heterogeneity at the gene expression level among individual lung metastases of a dedifferentiated chondrosarcoma patient. Despite this heterogeneity, we identified a set of “multifunctional” genes that are commonly expressed in the metastases. Also for the first time, we documented massive macrophage infiltration in the dedifferentiated chondrosarcoma lung metastases. It remains to be determined if the same phenomena will be observed in lung metastases of other dedifferentiated chondrosarcoma patients. Albeit derived from a single case, our findings have shed some light to the molecular mechanisms underlying metastasis in dedifferentiated chondrosarcoma.

## Supplementary Material

Supplemental Table 1: The differential expression of a subset of genes in the “biased multifunctional signature” was validated by quantitative real-time PCR. The Pfaffl method was used to calculate the relative gene expression levels.
Supplemental Table 2: Datasets for the functional analysis: Up or Down-regulated genes in all the Met.- cell lines, Multi-functional genes “commonly” expressed in the metastases and in the virtual non-metastatic tumor, Metastasis associated literature (GeneGo), “Multi-functional” grid (GeneGo).
Supplemental Table 3: Distribution of the SAGE tags cluster sizes that were significantly differentially expressed relative to those SAGE tags that did not exhibit differential expression in the metastases.
Supplemental Table 4: A. Pair-wise comparisons between the genes expressed in Met. 1 (4,205 genes) and those expressed in each of the other metastases, i.e. Met. 2–5. B. Similar pair-wise comparisons were performed between the subset of 105 “metastasis-associated” genes identified in Met. 1 and those expressed in each of the other metastases. C. Similar pair-wise comparisons were performed between the subset of 52 “Multi-functional” genes identified in Met. 1 and those expressed in each of the other metastases.
Supplemental Table 5: A. Expression of chondrocyte markers and markers of conventional chondrosarcoma in the metastases and the virtual non-metastatic tumor. B. Genes with altered expression in the dedifferentiated chondrosarcoma metastases, which also differentially expressed during chondrogenic differentiation of MSC. C. Evaluation of tumor cell invasion of the metastatic and non-metastatic cell lines.
Supplemental Figure 1: The network built for the “biased” multi-functional signature using shortest path (SP) algorithm. SP algorithm allows incorporation of certain nodes from the MetaCore database, which are not in the input list of 15 genes, in such a way that all the 15 input genes are connected by the smallest possible number of direct interactions. Red solid circle indicates expression of the gene in the metastases. Blue square indicates consistently down-regulated gene in the metastases, relative to the virtual NM-cell line.

## Figures and Tables

**Table 1:** Panel of SAGE libraries.

Library name	Tumor location	SAGE tags	Pathology
Met.-cell lines			
UIFK0 (Met. 1)	Right middle lobe, upper anterior metastatic lesion	87957	Dedifferentiated chondrosarcoma lung metastases
UIGP0 (Met. 2)	Right upper lobe, apical lateral metastatic lesion	108331	
UIGD0 (Met. 3)	Right lower lobe, middle diaphragmatic metastatic lesion	109862	
UIEK0 (Met. 4)	Right lower lobe, middle posterior metastatic lesion-	83522	
UIGX0 (Met. 5)	Left lower lobe, diaphragmatic anterior metastatic lesion	88439	
(NM-) cell lines			
UIGE0 (NM. 1)	Interior medial aspect of the pelvis. Fibrocartilaginous sample	77757	Recurrent nonmetastatic chondrosarcoma, grade 2
UIFU0 (NM. 2)	Interior medial aspect of the pelvis. Cartilaginous sample	77019	

**Table 2:** “Biased multifunctional signature” of dedifferentiated chondrosarcoma lung metastases.

Upregulated in Met.-cell lines	
Gene name	Gene symbol
Plasminogen activator, Tissue	PLAT*
Plasminogen activator, Urokinase	PLAU*
Interleukin 8	IL8*
Chemokine (C-C motif) ligand 2	CCL2
Integrin, beta 1	ITGB1
Actin, beta	ACTB
Vinculin	VCL
Drebrin 1	DBN1*
Moesin	MSN*
Tissue factor pathway inhibitor 2	TFPI2*
Caveolin 1	CAV1
Caveolin 2	CAV2*
Tenascin-C	TNC*

Downregulated in Met.-cell lines	

Transforming growth factor, beta-induced, 68 kDA	TGFBI
Serpin peptidase inhibitor, clade E, member 2	SERPINE2

*Genes uniquely expressed in the metastases.
